# Open Access as a Launchpad: Educational Pathways in Early-Career Scientific Publishing

**DOI:** 10.7759/cureus.103724

**Published:** 2026-02-16

**Authors:** José Emiliano González Flores

**Affiliations:** 1 Medicine, Tecnológico de Monterrey Campus Ciudad de Mexico, Mexico City, MEX

**Keywords:** academic authorship, citation impact, early-career researchers, open access publishing, peer review, publication pathways, research mentorship, research training, scholarly communication, scientific publishing

## Abstract

Open access publishing has transformed the scientific dissemination landscape by expanding accessibility and accelerating knowledge exchange. Beyond democratizing readership, it may also serve as a formative platform for early-career researchers navigating the complexities of academic authorship and scholarly communication. High rejection rates, prolonged review timelines, and limited mentorship exposure often position traditional publishing pathways as barriers to entry for young investigators. In contrast, engagement with open access journals can facilitate experiential learning in manuscript development, peer-review processes, and editorial interaction. Participation in accessible publication environments may support the acquisition of technical competencies, authorship confidence, and academic visibility during formative stages of scholarly development. This article is presented as a reflective editorial perspective rather than an empirical or systematic evaluation, aiming to conceptualize open access publishing as a progressive educational pathway within early-career scientific training. When approached critically and supported by structured mentorship, open access publishing functions not only as a dissemination model but also as a catalyst for the professional development of emerging scientific contributors.

## Editorial

Scientific publishing is widely regarded as the ultimate validation of academic productivity and research competence. However, for early-career researchers, the publication process often represents an entry barrier rather than a culmination of training. High rejection rates, prolonged review timelines, and limited exposure to editorial processes can make publishing appear inaccessible to young investigators. In this context, open access publishing may function not only as a dissemination model but also as a formative academic training platform [1].

The modern publication landscape is increasingly competitive. Early-career researchers frequently face repeated cycles of submission, rejection, and resubmission while targeting high-impact journals. These processes may delay academic progression and contribute to discouragement during critical stages of career development [2]. Consequently, accessible publication pathways play an important role in facilitating early scholarly engagement.

Open access journals offer a structurally different entry point into scientific publishing. By removing readership paywalls, they expand knowledge dissemination while simultaneously broadening authorship opportunities. More importantly, the process of preparing and submitting manuscripts provides experiential learning. Participation in research and publication activities has been associated with the development of transferable competencies, including scientific writing, data interpretation, and methodological reasoning [3].

From a personal academic perspective, open access publishing has also functioned as a formative training ground. Early in my research career, engaging with accessible journals allowed me to understand the mechanics of manuscript structuring, editorial workflows, and peer-review dynamics while navigating my own learning curve as an author. These initial experiences did not represent endpoints of scientific productivity but rather structured stages of academic development. Through iterative submission, revision, and editorial dialogue, open access environments facilitated the acquisition of practical competencies that formal research curricula rarely provide.

From a training perspective, early publication experiences function as practical rehearsal environments. Young researchers learn how to structure manuscripts, respond to peer review, and navigate authorship collaboration. However, beyond technical writing, these interactions cultivate editorial literacy, the ability to interpret reviewer intent, negotiate revisions, and refine scientific argumentation. Accessible publishing platforms often provide earlier exposure to these processes, allowing investigators to develop procedural confidence before engaging with higher-selectivity journals. In this sense, open access publishing contributes not only to dissemination but to the professional maturation of emerging researchers.

Skepticism persists regarding the scientific impact of open access journals. Concerns frequently focus on peer-review rigor and citation performance. Comparative bibliometric analyses suggest that such differences diminish when controlling for discipline, journal maturity, and indexing status, particularly within biomedical fields [4]. These findings challenge the perception that open access publication inherently reflects lower scientific influence.

A critical distinction within the open access landscape lies between predatory or substandard publication venues and legitimate, peer-reviewed journals that adhere to recognized indexing, editorial transparency, and ethical publishing standards. For early-career researchers, navigating this distinction represents both a challenge and a formative learning opportunity. Engagement with open access publishing should therefore be approached critically, emphasizing due diligence in journal selection, verification of indexing status, peer-review integrity, and adherence to publication ethics. Structured mentorship plays a central role in this process, guiding novice authors in identifying credible journals, interpreting impact and visibility metrics, and avoiding exploitative publishing practices. When supported by informed supervision, early publication experiences within reputable open access environments can preserve scientific rigor while still functioning as accessible training platforms. In this context, the educational value of open access publishing is not inherent to the model itself but contingent upon responsible navigation, institutional guidance, and ethical journal targeting.

Financial considerations remain an important structural variable within the open access training pathway. Article processing charges (APCs), while sustaining publication infrastructure, may simultaneously introduce access inequities, particularly for early-career researchers and investigators from low-resource or underfunded institutional environments. These financial barriers can influence journal selection, submission frequency, and the overall feasibility of early publishing engagement. Nonetheless, institutional support mechanisms, including APC waivers, mentorship-guided journal targeting, and collaborative authorship models, may mitigate these constraints and preserve equitable participation. The question of whether early open access publication predicts long-term academic productivity or grant success remains empirically heterogeneous. While preliminary bibliometric observations suggest that early publication exposure may enhance authorship confidence, scholarly visibility, and research continuity, causality is difficult to establish given the multifactorial determinants of academic advancement [3,4,5]. Framing open access publishing as a developmental pathway should therefore be interpreted not as a deterministic predictor of career success, but as an enabling environment that may facilitate progressive scholarly maturation when supported by mentorship and institutional scaffolding.

If open access publishing is to be operationalized as a developmental training pathway, structured mentorship practices must accompany early publication exposure. Institutions and senior investigators can facilitate this process through guided journal vetting workshops, supervised manuscript development, and peer-review simulation exercises that familiarize trainees with editorial expectations. Additional practices may include collaborative authorship models, staged writing feedback, and formal instruction in publication ethics, authorship criteria, and indexing standards. Such frameworks transform early publishing from an isolated technical activity into a supervised educational continuum. By embedding publication training within mentorship structures, early-career researchers may engage with open access environments critically, ethically, and strategically, maximizing educational value while preserving scientific integrity.

The question of citation advantage remains debated. Systematic evaluations comparing open access and subscription-based publications report heterogeneous outcomes. Some studies demonstrate increased citation rates, while others show no difference or advantages limited to specific disciplines [5]. Rather than undermining open access credibility, this variability underscores the multifactorial determinants of scientific impact.

Conceptualizing publishing as a progressive pathway may be especially beneficial for early-career researchers. Initial experiences in accessible journals can build technical competence, authorship confidence, and academic visibility. These formative steps may prepare investigators for subsequent engagement with more selective speciality and high-impact journals. In this model, open access functions as a launchpad rather than a terminal destination.

Recognizing the pedagogical value of open access publishing carries implications for mentors and academic institutions. Structured mentorship remains essential in this process. Guidance in journal selection, indexing standards, peer-review transparency, and publication ethics ensures that early publishing experiences remain constructive. When paired with responsible mentorship, accessible publishing platforms evolve from simple dissemination mechanisms into supervised academic training pathways.

Scientific advancement depends not only on discovery but also on the capacity to train future investigators. Open access publishing, when navigated critically and responsibly, does not dilute scientific rigor, it cultivates the very researchers who will sustain it. Rather than viewing accessible journals as secondary arenas of science, the academic community should recognize them as developmental platforms where competence, confidence, and credibility begin to take shape.
